# The impact of “Option B” on HIV transmission from mother to child in Rwanda: An interrupted time series analysis

**DOI:** 10.1371/journal.pone.0192910

**Published:** 2018-02-16

**Authors:** Monique Abimpaye, Catherine M. Kirk, Hari S. Iyer, Neil Gupta, Eric Remera, Placidie Mugwaneza, Michael R. Law

**Affiliations:** 1 Rwanda Biomedical Center, Kigali, Rwanda; 2 Partners In Health/Inshuti Mu Buzima, Rwinkwavu, Rwanda; 3 Brigham and Women’s Hospital, Boston, MA, United States of America; 4 The University of British Columbia, Vancouver, British Columbia, Canada; 5 University of Rwanda, Kigali, Rwanda; 6 Harvard Medical School, Boston, MA, United States of America; University of Massachusetts Amherst, UNITED STATES

## Abstract

**Background:**

Nearly a quarter of a million children have acquired HIV, prompting the implementation of new protocols—Option B and B+—for treating HIV+ pregnant women. While efficacy has been demonstrated in randomized trials, there is limited real-world evidence on the impact of these changes. Using longitudinal, routinely collected data we assessed the impact of the adoption of WHO Option B in Rwanda on mother to infant transmission.

**Methods:**

We used interrupted time series analysis to evaluate the impact of Option B on mother-to-child HIV transmission in Rwanda. Our primary outcome was the proportion of HIV tests in infants with positive results at six weeks of age. We included data for 20 months before and 22 months after the 2010 policy change.

**Results:**

Of the 15,830 HIV tests conducted during our study period, 392 tested positive. We found a significant decrease in both the level (-2.08 positive tests per 100 tests conducted, 95% CI: -2.71 to -1.45, p < 0.001) and trend (-0.11 positive tests per 100 tests conducted per month, 95% CI: -0.16 to -0.07, p < 0.001) of test positivity. This represents an estimated 297 fewer children born without HIV in the post-policy period or a 46% reduction in HIV transmission from mother to child.

**Conclusions:**

The adoption of Option B in Rwanda contributed to an immediate decrease in the rate of HIV transmission from mother to child. This suggests other countries may benefit from adopting these WHO guidelines.

## Introduction

Globally, nearly a quarter of a million children have acquired HIV, the vast majority in sub-Saharan Africa.[1] This has prompted the development and implementation of new protocols for treating HIV-positive pregnant women for prevention of mother to child transmission (PMTCT). The provision of triple antiretroviral therapy (ART) through the pregnancy and breastfeeding period—known as “Option B” as designated by the World Health Organization (WHO),[2]—has been shown in clinical trial settings to be more efficacious than single-drug prophylaxis for mother and infant through six weeks, plus ongoing prophylaxis for the infant through breastfeeding, otherwise known as “Option A”, for PMTCT.[3] Other clinical trials and small-scale observational studies have corroborated this finding in sub-Saharan Africa.[4–7]

As a result of these findings and the evidence of benefits of early ART treatment,[8, 9] the WHO has called for global adoption of Option B+, which extends Option B treatment to all mothers with HIV for life independent of immune status.[10] However, challenges in implementation of Option B+ remain, including human resource constraints, low service utilization, and poor retention in care.[11–13] Further, the benefits of triple ART for PMTCT may be overestimated by clinical trial findings given sub-optimal implementation in real-world settings.[14]

There is limited population-level evidence on the benefit of Option B/B+ on transmission of HIV from mother to child in national-scale, real-world implementation in the literature.[15] Given the large monetary and resource investment required for providing triple ART for all HIV-positive pregnant women, particularly for life,[16–19] it is critical to ascertain whether the benefits observed in clinical trial settings are replicated in real-world settings. The availability of routinely collected data in Rwanda allowed for a unique opportunity to conduct an impact evaluation at national-scale. Using national reporting systems, we studied the impact of Rwanda’s adoption of Option B/B+ on transmission of HIV from mother to child.

## Materials and methods

### Program setting

Rwanda implemented Option B in November 2010, making it one of the first countries in sub-Saharan Africa to do so.[18] Option B+ was quickly thereafter adopted in April 2012, prior to the programmatic cessation of ART for any pregnant women who were started under Option B. [20] Prior to 2010, Rwanda was providing ART to mothers during pregnancy (zidovudine/AZT), delivery (single-dose nevirapine), and for one week after delivery (AZT and lamivudine/3TC), and infants received single-dose nevirapine at birth in addition to one month of AZT following delivery [21]; this regimen used in Rwanda was slightly different than WHO’s proposed Option A in the 2010 PMTCT Guidelines, which consisted of a single-drug regimen for the mother (AZT) throughout pregnancy with either AZT or nevirapine for the infant for 6 weeks or until one week after the end of the breastfeeding period (depending on which was longer).[22] Rwanda’s PMTCT program is integrated into antenatal care services, with HIV testing offered to all pregnant women and their partners at their first antenatal visit. PMTCT services are provided primarily by trained nurses at health centers, which are responsible for routine primary care in Rwanda,[23] and were available at 85% of health facilities at the time Option B was implemented.

### Data source and outcome

Our analysis used data from the national HIV reporting database (TRACnet), a web-based health facility reporting system for HIV services in Rwanda. Facilities report aggregate data into TRACnet on a monthly basis from patient registers completed by nurses. TRACnet has undergone routine data quality assessments since 2007 and has been used in prior studies.[24] We included all health facilities with complete monthly reporting for the entire study period from June 2009 to November 2012 (n = 238/343, or 69% facilities that provided PMTCT services). Our primary outcome was the HIV transmission rate from mother to child at 6 weeks, which we calculated as the number of positive tests per 100 HIV tests conducted each month.

### Study design and analysis

We assessed the real-world impact of Rwanda’s implementation of Option B/B+ on HIV transmission from HIV-positive mothers to infants at six weeks using routinely collected, national-level data. Option B was adopted and implemented beginning in Nov 2010 and Option B+ in April 2012. To allow for scale-up and viral load suppression of women newly started on combination ART, we assessed the impact of the policy after a three-month window had elapsed. Thus, we established the intervention start date for the interrupted time series analysis in February 2011, three months after the policy change in November 2010.

We used interrupted time series analysis to study trends in monthly HIV transmission from mother to child at six weeks after birth before and after the implementation of Option B.[25, 26] Our interrupted time series models were fit using segmented regression models and longitudinal data. In our model, we investigated possible autocorrelation between data points in our generalized least squares regression using the Durbin-Watson test and autocorrelation plots. Interrupted time series analysis is an ideal, robust method for policy analysis.[27] This method longitudinally models shifts in both the level and trend in an outcome after a policy change, in this case the adoption of Option B. Our data period provided us with 20 monthly observations prior to the adoption of Option B (June 2009-January 2011) and 22 monthly observations afterward (February 2011-November 2012). Based on tests for autocorrelation, we fit segmented regression models using generalized least squares regression including a fifth-order autoregressive correlation structure. Pre-intervention level and linear trends were plotted using the model coefficients and counterfactual values of the level and trend calculated for comparisons.[28]

### Ethical considerations

This study analyzed routine anonymous, facility-level data collected and reported monthly by the facilities that provide PMTCT services though the TRACnet reporting system and was exempt from ethical review.

## Results

Over the study period, there were 15,830 six-week HIV tests conducted among infants born to HIV-positive mothers, 392 of which tested positive. In the 20 months prior to February 2011, 7,310 six-week tests were conducted (mean = 365.5 per month, SD = 42.7) and 242 infants tested positive (mean = 12.1 per month, SD = 7.2). In the 22 months after the policy change, 8,520 six-week tests were conducted (mean = 387.3 per month, SD = 39.3) and 150 infants tested positive (mean = 6.8 per month, SD = 3.7). On average, the transmission rate decreased from 3.3% prior to the policy change to 1.8% following it, which is a 46% relative decrease in HIV transmission following the adoption of Option B.

**Fig 1:**
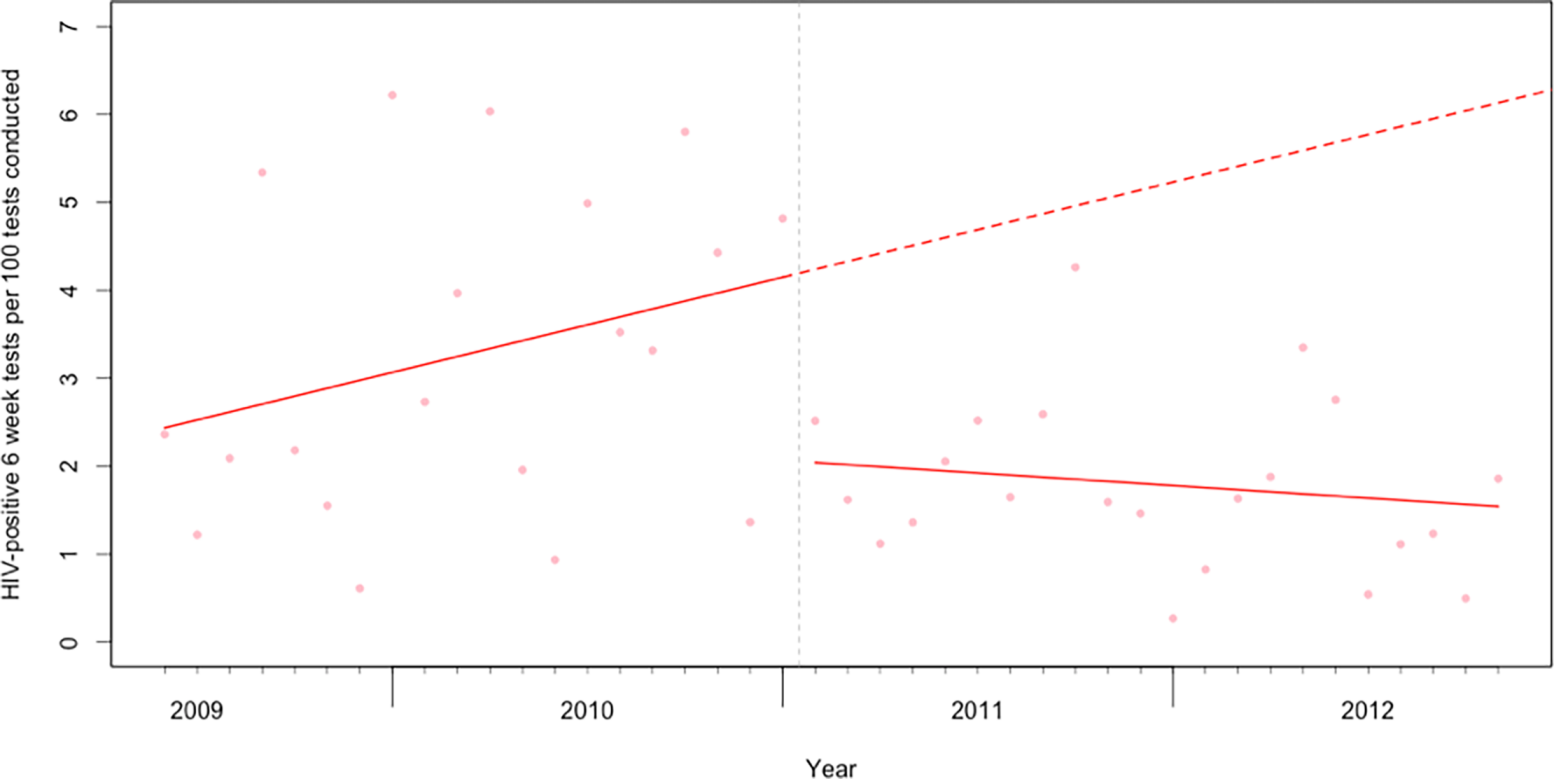
The rate of HIV-positive six-week tests per 100 tests conducted prior to and following the implementation of Option B in Rwanda. The dashed vertical line represents the first month in which we expected to see the results of Option B adoption (February 2011, 3 months after the implementation of Option B in November 2010). The solid red lines show the fitted values from the interrupted time series model, and the dashed red line the predicted counterfactual trend absent the policy change.

Our interrupted time series analysis found a significant immediate decrease of -2.08 HIV-positive results per 100 tests conducted following the implementation of Option B (95% CI: -2.71 to -1.45, p < 0.001, see Fig 1). We also found a decrease in the trend of -0.11 in each month thereafter (95% CI: -0.16 to -0.07, p < 0.001). Overall, this represents approximately 297 fewer infants that tested positive for HIV at six weeks following the implementation of Option B at these health facilities over the course of our study period.

## Discussion

Using a rigorous, longitudinal study design and comprehensive administrative data, we found a decrease in the number of infants who tested positive for HIV six weeks after birth following the adoption of WHO Option B. We found a 66% reduction in the number of positive six-week tests compared to the expected number given existing pre-policy trends. This is comparable to initial results from the PROMISE clinical trial showing a 67–72% reduction in transmission rates.[3] This similarity indicates that the real-world impact of adopting Option B can be similar to the effects observed in clinical trials, and provides backing for the use of administrative data systems such as TRACnet in the evaluation of such policy changes. We also observed a significant decreasing trend of HIV transmission at six weeks overtime after the implementation of Option B, which may be the result of better execution of Option B overtime by providers ensuring that all women testing positive received the correct regimen under Option B.

Other contextual factors in addition to the change in the PMTCT protocol may have contributed to our observed decline in HIV transmissions. The rapid scale up of PMTCT sites provided improved proximity and accessibility to these services, however these changes occurred primarily before the study period with national availability of PMCT services at health facilities remaining steady around 80% of facilities during the period of study.[29] Further, Rwanda’s implementation of Option B occurred among other interventions to strengthen the health system and improve quality of service delivery. For instance, there have been ongoing and consistent increases in the percentage of women with comprehensive knowledge on HIV transmission, delivering in a facility, and utilizing contraceptive methods.[30] However, these improvements have been gradually occurring over time, and thus would not bias our interrupted time series analysis as they would not have induced abrupt changes in our outcome at the same point as the change to Option B. The increasing trend in HIV six-week transmission rates prior to the switch to Option B was a surprising finding, but has been documented elsewhere [29]. The cause of this trend is not known, however the complexity of the regimen prior to Option B, improving laboratory capacity overtime, and variability in monthly transmission rates may have contributed to it.

We note some important limitations to our work. First, we did not have access to the total number of HIV-positive mothers in our dataset. However, given that 98% of women in Rwanda are tested for HIV during pregnancy[30] and that an estimated 89% of HIV-positive pregnant women receive ART for PMTCT,[31] we believe that using six-week tests as a denominator is appropriate. Second, data on the results of subsequent HIV tests at nine months and 18 months was not available, so we cannot comment on the impact of this policy on longer-term transmission. Finally, we note that our data is quite variable, particularly in the pre-intervention period, which may have led to the positive trend in six-week transmissions from 2009–2010. We believe this variability may represent prolonged delays and fluctuations in result turn-around time in those years, resulting in more dramatic variability across months.[29] However, as our models examine trends and account for autocorrelation, which may be due to factors such as seasonal variability in access to care, this variability would only act to make our results more statistically conservative as it would have made our observed standard errors larger. Lastly, the transmission rates from mother to child on the regimen used in Rwanda prior to adoption of Option B are not well studied and to our knowledge there is no data available to assess whether the transmission rates under the regimen prior to Option B were within the expected range.

## Conclusion

These results suggest that the adoption of Option B/B+ contributed to a national decline in HIV transmission to children at six weeks following birth in Rwanda. These findings provide population-level evidence that support WHO recommendations for wide-scale adoption and implementation of Option B+ in sub-Saharan Africa.[10]
